# Antioxidant and tyrosinase inhibition activity of the fertile fronds and rhizomes of three different Drynaria species

**DOI:** 10.1186/s13104-015-1414-3

**Published:** 2015-09-22

**Authors:** Joash Ban Lee Tan, Yau Yan Lim

**Affiliations:** School of Science, Monash University Malaysia, Bandar Sunway, 46150 Petaling Jaya, Selangor Malaysia

**Keywords:** Fern rhizome, Fertile fronds, Fern leaves, Free radical scavenging, Tyrosinase inhibition

## Abstract

**Background:**

For generations, the rhizomes of Drynaria ferns have been used as traditional medicine in Asia. Despite this, the bioactivities of Drynaria rhizomes and leaves have rarely been studied scientifically.

**Methods:**

This study evaluates the antioxidant properties of the methanolic extracts of the fertile fronds and rhizomes from three species in this genus: *Drynaria quercifolia*, *Drynaria rigidula* and *Drynaria sparsisora*. The phenolic and flavonoid contents of the samples were respectively quantified with the total phenolic content (TPC) and total flavonoid content (TFC) assays, while the antioxidant activities were determined via measuring the DPPH radical scavenging activity (FRS), ferric reducing power (FRP), ferrous ion chelating (FIC) activity and lipid peroxidation inhibition (LPI). The tyrosinase inhibition activity of all three species was also reported.

**Results:**

The fertile fronds of *D. quercifolia* were found to exhibit the highest overall TPC (2939 ± 469 mg GAE/100 g) and antioxidant activity amongst all the samples, and the fertile fronds of *D. quercifolia* and *D. rigidula* exhibited superior TPC and FRP compared to their rhizomes, despite only the latter being widely used in traditional medicine. The fronds of *D. quercifolia* had high tyrosinase inhibition activity (56.6 ± 5.0 %), but most of the Drynaria extracts showed unexpected tyrosinase enhancement instead, particularly for *D. sparsisora*’s fronds.

**Conclusion:**

The high bioactivity of the fertile fronds in the fern species indicate that there is value in further research on the fronds of ferns which are commonly used mostly, or only, for their rhizomes.

## Background

Interest in phenolic compounds has been on the rise due to their potential human health benefits [1]. They are the most abundant class of plant antioxidants—compounds capable of deactivating or stabilizing free radicals, thereby reducing free-radical-mediated cellular and tissue damage [2]. Plant antioxidants have been purported to have anti-aging properties, and may prevent numerous diseases such as cancer, diabetes, neurodegenerative diseases [3], atherosclerosis, and cardiovascular diseases [4]. Some phenolic compounds are also able to inhibit tyrosinase, an enzyme responsible in melanogenesis and enzymatic browning. This has considerable commercial value in the cosmetics industry as a skin-whitening agent, or an anti-browning agent in the food industry [5]. Tyrosinase inhibition has also been discovered to reduce the viability of catecholaminergic neuronal cells [6] which have been linked to several psychiatric and neurodegenerative disorders, thus providing a possible new treatment in the future [7].

The rhizomes of the Drynaria genus of ferns have a long history of being used as traditional medicine, particularly in India [8], China [9] and Southeast Asia [10]. *Drynaria quercifolia* (L.) J. Smith is one of the best-known members of this genus, commonly used in Ayurvedic medicine (“Ashwakatri”) [8] where its boiled rhizome decoction is consumed orally for its anti-pyretic properties; and used as a treatment for tuberculosis [10], diarrhea, cholera, fever, typhoid, syphilis and skin diseases [11]. Additionally, the extract of this fern was capable of inhibiting wildtype and multidrug-resistant bacteria such as *Neisseria gonorrhoeae* and *Streptococcus*-*β*-*haemolyticus* [10]. Due to the ethnobotanical value of the *D. quercifolia*’s rhizomes, it has been the emphasis of phytochemical-centric research on the species. In comparison, little research has been conducted on the fertile fronds (leaves), which are sometimes used in conjunction with the rhizome to treat tuberculosis and throat infections [12], or as a poultice to reduce swelling [8].

The rhizomes of *Drynaria rigidula* (Sw.) Beddome and *Drynaria sparsisora* (Desv.) T. Moore have also been claimed to treat similar diseases as the rhizomes of *D. quercifolia,* such as diarrhea and gonorrhea [13]. However, unlike *D. quercifolia,* there have been no reports on the bioactivity of the fertile fronds of both species. *D. rigidula* is an endangered species in many locations [14], while *D. sparsisora* is nearly identical to *D. quercifolia* in physical appearance, but with shorter fertile fronds [15] and a dark-colored scaly rhizome [16]. This study represents the first time that the antioxidant activities of *D. rigidula* and *D. sparsisora* fertile fronds and rhizomes have ever been reported, and the comparison between the antioxidant activity of fertile fronds and rhizomes in a fern remains a rarely-explored avenue in most literature. This is also the first time that the tyrosinase inhibition activity for any of these species has ever been reported.

## Methods

### Collection of fern samples

The fertile fronds and rhizomes of *D. quercifolia* and *D. rigidula* were obtained from the Putrajaya Botanical Garden, Kuala Lumpur, while the leaves and rhizomes of *D. sparsisora* were obtained from Sunway, Petaling Jaya. The ferns were identified by plant taxonomist Anthonysamy S., formerly from University Putra Malaysia.

### Chemicals and reagents

The various reagents used throughout this project were purchased from suppliers as follows. TPC analysis: Folin–Ciocalteu’s phenol reagent (2 N, R and M Chemicals, Essex, UK), gallic acid (98 %, Fluka, Steinheim, France), anhydrous sodium carbonate (99 %, J. Kollin, UK); total flavonoid content (TFC) analysis: aluminium chloride (99.5 %, Bendosen Laboratory Chemicals, Bendosen, Norway), potassium acetate (99 %, R and M chemicals), quercetin (98 %, Sigma St. Louis, MO, USA); diphenyl-2-picrylhydrazyl (DPPH·) assay: 1,1-diphenyl-2-picrylhydrazyl (90 %, Sigma, St. Louis, MO, USA); ferric reducing power (FRP) assay: ferric chloride hexa-hydrate (100 %, Fisher Scientific, Loughborough, UK), potassium ferricyanide (99 %, Unilab, Auburn, Australia), trichloroacetic acid (99.8 %, HmbG Chemicals, Barcelona, Spain), potassium dihydrogen orthophosphate (99.5 %, Fisher Scientific, Loughborough, UK), dipotassium hydrogen phosphate (99 %, Merck, Darmstadt, Germany), iron chloride (99 %, R&M Chemicals, Petaling Jaya, Malaysia); ferrous ion chelating (FIC) assay: ferrozine (98 %, Acros Organics, Morris Plains, NJ, USA), ferrous sulphate hepta-hydrate (HmbG Chemicals, Barcelona, Spain), ethylenediaminetetraacetic acid (EDTA) (98 %, Sigma, St. Louis, MO, USA); lipid peroxidation inhibition (LPI): β-carotene (Sigma, St. Louis, MO, USA), chloroform (Fisher Scientific, 99.9 %, Loughborough, UK), linoleic acid, C_18_H_32_O_2_ (Fluka, Steinheim, France), Tween 40 (Fluka, Steinheim, France); tyrosinase inhibition activity: 3, 4-dihydroxy-l-phenylalanine C_9_H_11_NO_4_ (Sigma, St. Louis, MO, USA), dimethyl sulfoxide analytical grade (Fisher Scientific, Loughborough, UK), kojic acid C_6_H_6_O_4_ (Sigma, St. Louis, MO, USA), tyrosinase (catechol oxidasemonophenol, dihydroxyphenylalanine) 3400 units/mg solid (Sigma, St. Louis, MO, USA).

### Extraction of samples

Fresh fertile fronds were extracted at a ratio of 50 mL 70 % methanol to 1 g of leaf material after liquid nitrogen-aided crushing. For the rhizomes, 1.0 g of de-skinned rhizome was extracted at a ratio of 50 mL 50 % methanol to 1 g of rhizome. The methanol concentrations chosen for extraction were based on preliminary extraction efficiency screening, where 70 and 50 % methanol were found to be more efficient at extracting the fertile fronds and rhizomes respectively. The extracts were then filtered with a Buchner funnel. The methanolic extracts were stored at −20 °C when not in use.

### Determination of antioxidant activity

#### Determination of total phenolic content (TPC)

The determination of the total phenolic content of the samples was done using a procedure modified from Kähkönen et al. [17] utilizing the Folin–Ciocalteu reagent. Samples (300 μL, in triplicate) were mixed with 1.5 mL of the 10 % Folin–Ciocalteu reagent, followed by an addition of 1.2 mL of 7.5 % (w/v) sodium carbonate (Na_2_CO_3_) solution. The test tubes were then left to stand for 30 min in the dark at room temperature before the absorbance values were measured at 765 nm. The total phenolic content was expressed as mg gallic acid equivalent per 100 g of sample (mg GAE/100 g).

#### Total flavonoid content (TFC)

Flavonoid content in the extract was determined with the aluminium chloride colorimetric method as described in Chang et al. [18]. Equal volumes of 10 % aluminium chloride and 1.0 M potassium acetate (0.1 mL each) were added to 0.5 mL of extract, followed by 2.8 mL of distilled water. The solutions were mixed well and incubated at room temperature for 30 min before the absorbance was taken at 415 nm. The flavonoid concentration was expressed as mg quercetin equivalent per 100 g sample, mg QE/100 g.

#### DPPH radical scavenging assay (FRS)

The DPPH· assay was based on the procedures described in Leong and Shui [19] and Miliauskas et al. [20] where the reduction of the DPPH (2,2-diphenyl-1-picrylhydrazyl) radical was measured spectrometrically to determine the radical scavenging activity of the extract. Two mL of DPPH· solution (5.9 mg in 100 mL methanol) was added to 1 mL of three different concentrations of the of sample extract (diluted with methanol). The absorbance of the solution was measured at 517 nm after a 30 min incubation time. The free radical scavenging activity (FRS) was expressed as ascorbic acid (AA) equivalent antioxidant capacity, in mg AA/100 g using the equation: ${\text{AEAC}} = {\text{IC}}_{{ 50({\text{AA}})}} /{\text{IC}}_{{ 50({\text{sample}})}} \; \times \; 10^{ 5}$. IC_50_ of AA used for calculation of FRS was 0.00387 mg/mL.

#### Ferric reducing power (FRP) assay

The reducing power of the extracts was determined using potassium hexacyanoferrate(III) as described in the procedure described by Tan and Chan [21]. The FRP assay was used to assess the ability of any antioxidants present in the extracts to reduce ferric ions (Fe^3+^) to ferrous ions (Fe^2+^). One mL of sample extract of different concentration (diluted with methanol) was added with 2.5 mL of 0.2 M phosphate buffer (pH 6.7) and the same volume of 1 % (w/v) potassium ferricyanide. The solutions were mixed and incubated in 50 °C water bath for 20 min. Subsequently, 2.5 mL of 10 % trichloroacetic acid was added to stop the reaction. Then, the solution in each test tube was separated into aliquots of 2.5 mL, added with 2.5 mL of Milli-Q water and 0.5 mL of 0.1 % FeCl_3_. The solutions were mixed and left on bench for 30 min before the absorbance was measured at 700 nm. FRP was expressed as mg gallic acid equivalent per gram of sample, mg GAE/g.

#### Ferrous ion chelating (FIC) assay

The determination of ferrous ion chelating strength of the extract was based on the procedures described in Mau et al. [22], and Singh and Rajini [23]. One mL of 0.1 mM FeSO_4_ was added to 1 mL of sample of different concentrations (0.2, 0.5 and 1 mL of extract, diluted with methanol), followed by 1 mL of 0.25 mM ferrozine. The mixtures were incubated at room temperature for 10 min before the absorbance was measured at 562 nm. It was expressed as the percentage of iron chelating activity. EDTA (0.017–0.067 mg/mL) was used as a positive control.

#### Lipid peroxidation inhibition (LPI)

Lipid peroxidation inhibition was adapted from Kumazawa et al. [24] based on the bleaching of β-carotene with slight modifications. Six mg of β-carotene was dissolved in 50 mL of chloroform, and 4 mL of the solution was mixed with 40 mg of linoleic acid and 400 mg of Tween 40 emulsifier in a conical flask. Nitrogen gas was then used to evaporate the chloroform. Then, 100 mL of oxygenated Milli-Q water was added and the flask was shaken for the mixture to be fully dissolved. Immediately after water was added, the absorbance of the mixture was measured at 470 and 700 nm using Perkin-Elmer double-beam spectrophotometer. Next, 3 mL of the emulsion was added into test tubes with different volume of sample (10, 50 and 100 μL). The tubes were sealed with parafilm and incubated at 50 °C water bath for an hour before the absorbance was measured again. For control, 100 μL of methanol was used instead of the sample. The blank was prepared by adding the same volume of sample with the emulsion of 400 mg Tween 40 emulsifier and 100 mL of water but without linoleic acid and β-carotene solution. The absorbance (A) at 700 nm was taken in order to correct the haze present in the solution. The LPI was calculated using the formula:

$${\text{Degradation Rate }}\left( {\text{DR}} \right) \, = \, \left[ {{\text{Ln }}\left( {{\text{A}}_{\text{Initial}} / {\text{A}}_{\text{Sample}} } \right)} \right]/ 60$$

$${\text{Antioxidant Activity }}\left( {\% {\text{ AOA}}} \right) \, = \, \left[ { 1 { }{-} \, \left( {{\text{DR}}_{\text{Sample}} / {\text{DR}}_{\text{Control}} } \right)} \right]\; \times \; 100.$$

### Determination of tyrosinase inhibition activity

The measurement of tyrosinase inhibition activity of extracts was based on the method used by Masuda et al. [25], with slight modification. The method utilizes l-DOPA and a 96-well microplate for screening of multiple samples simultaneously. For testing of the sample extract, the wells in triplicate were added with 80 μL of buffer (0.1 M phosphate buffer, pH 6.8), 40 μL of tyrosinase (1 mg/mL of tyrosinase diluted 50-folds with Milli-Q water), and 40 μL of sample (1 mg of freeze-dried extract in 400 μL of 50 % DMSO), and lastly 40 μL of l-DOPA. For the control, the same reagents were added to the wells except that the sample was substituted with 40 μL of DMSO. The blank contained 120 μL of buffer, 40 μL of tyrosinase, and 40 μL of sample. The mixture in each well was mixed and left on the bench for 30 min (after the addition of l-DOPA). Subsequently, the microplate was measured using BIOTEK PowerWave XS Microplate Scanning Spectrophotometer at the absorbance of 475 nm, with reference wavelength at 700 nm. The percentage tyrosinase inhibition activity was calculated using equation below:

$$\% {\text{ Tyrosinase inhibition activity}} = \, \left[ { 1 { }{-} \, \left( {{\text{A}}_{\text{Sample}} / {\text{A}}_{\text{Control}} } \right)} \right]\; \times \; 100.$$

### Statistical analysis

Statistical analysis was carried out with one-way ANOVA and the Tukey HSD test was used to identify any significance between the TPC values of the samples. *p* < 0.05 was considered to be significantly different.

## Results and discussion

The TPC, FRS, FRP and TFC results reported in Table 1 are a measure of the primary antioxidant activity—the ability to scavenge free radicals, thus inhibiting chain initiation and terminating chain propagation [26]. *D. quercifolia* fertile fronds exhibited a very high TPC of 2939 ± 469 mg GAE/100 g—consistent with previous findings reported by our research group where *D. quercifolia* fertile fronds ranked second highest in TPC amongst the fifteen ferns screened with an average TPC exceeding 2500 mg GAE/100 g [27]. *D. quercifolia* had the highest TPC of the three species screened: approximately three times higher than the fronds of *D. rigidula* and nearly ten-fold higher than the fronds of *D. sparsisora*; a surprising result given the similar physical appearance of the *D. quercifolia* and *D. sparsisora* fronds. The results for the FRS activity (IC_50_ and AEAC) reflected a similar correlation, with *D. quercifolia* fronds exhibiting the highest activity, followed by *D. rigidula* and *D. sparsisora*. However, despite the higher TPC and FRS in *D. rigidula* fronds compared to *D. sparsisora* fronds, the FRP between the fronds of both species were similar thus indicating that phenolic compounds may not necessarily be the only reducing agents present in the fronds of *D. sparsisora*.

**Table 1:** Total phenolic content (TPC), free radical scavenging activity (IC50 and ascorbic acid equivalent antioxidant capacity, AEAC), ferric reducing power (FRP) and total flavonoid content (TFC) of Drynaria sp fertile fronds and rhizomes

Species	Part	TPC (mg GAE/100 g)	IC_50_ (mg/mL)	AEAC (mg AA/100 g)	FRP (mg GAE/g)	TFC (mg QE/100 g)
*D. quercifolia*	Frond	2939 ± 469^a^	0.09 ± 0.01^a^	4456 ± 571^a^	17.5 ± 2.2^a^	0.1 ± 0.0^a^
	Rhizome	1732 ± 437^b^	0.17 ± 0.03^b^	2273 ± 415^b^	9.5 ± 1.3^b^	5.0 ± 0.1^b^
*D. rigidula*	Frond	1031 ± 132^c^	0.79 ± 0.22^c^	507.3 ± 151.0^c^	2.3 ± 0.3^c^	4.7 ± 0.9^b^
	Rhizome	305.5 ± 17.2^d^	1.02 ± 0.16^c^	380.6 ± 64.9^c^	1.0 ± 0.2^d^	3.1 ± 0.1^c^
*D. sparsisora*	Frond	316.6 ± 19.5^d^	2.01 ± 0.62^d^	199.9 ± 53.2^d^	2.1 ± 0.3^c^	1.0 ± 0.5^d^
	Rhizome	367.2 ± 20.9^d^	0.81 ± 0.18^c^	489.4 ± 126.5^c^	2.2 ± 0.6^c^	0.1 ± 0.1^a^

Results are expressed as mean ± SD (n = 3). For each column, values followed by the same letter are not significantly different at p < 0.05 as measured by the Tukey HSD test

When compared to the fertile fronds, the rhizomes of *D. quercifolia* and *D. rigidula* exhibited lower primary antioxidant activity, with lower TPC and weaker FRP activity (Table 1). Only in *D. sparsisora* was there no significant difference in TPC and FRP between the leaves and rhizomes, but the rhizomes showed significantly higher FRS than the fronds. Despite the TPC of *D. sparsisora*’s leaves and rhizomes being comparable with one another, the free-radical scavenging capability of the rhizomes was considerably higher than would be expected based on the TPC alone. This indicates the rhizomes of *D. sparsisora* either contain non-phenolic free-radical scavengers, or the phenols in the rhizomes are better free radical scavengers than those in the fertile fronds. While it has been reported that the leaves of a plant may contain more phenols than the rhizomes [28, 29], this is nevertheless an interesting finding as thus far, only the rhizomes of these ferns have been used in ethnobotany.

**Fig. 1:**
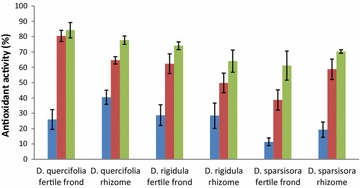
Lipid peroxidation inhibition (LPI) of fertile fronds and rhizomes of Drynaria sp. The figure shows *bars* of three *different colors*. The *blue bar* represents “66.4 μg/mL”, the *red bar* represents “327.9 μg/mL” and the *green bar* represents “645.2 μg/mL”

The lipid peroxidation inhibition (LPI) activity (Fig. 1), measures the ability of an antioxidant to inhibit lipid peroxidation, thus being a more accurate assessment of the hydrophobic antioxidants present. This is in contrast to the FRS activity (Table 1) that measures the antioxidant activity of both hydrophilic and hydrophobic compounds. All three species showed considerable antioxidant activity of 50 % or above at the maximum concentration tested (645.2 μg fresh sample/mL) in a concentration-dependant manner. The LPI between the fronds and rhizomes were comparable despite the differences in other facets of their antioxidant activity (Table 1), indicating the hydrophobic antioxidants present in both the fronds and rhizomes were comparable in antioxidant activity. Examples of such hydrophobic antioxidants would include terpenoids/carotenoids and tocopherols [30].

**Fig. 2:**
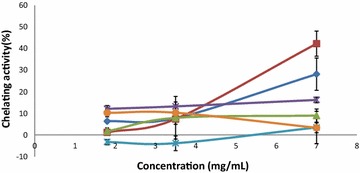
Ferrous iron chelating (FIC) activity in fertile fronds and rhizomes of Drynaria sp. The figure shows *six lines* with *different colors*. The *darker blue line* represents “*Drynaria quercifolia* fertile fronds”. The *red line* represents “*Drynaria rigidula* fertile fronds”. The *green line* represents “*Drynaria sparsisora* fertile fronds”. The *purple line* represents “*Drynaria quercifolia* rhizome”. The *lighter blue line* represents “*Drynaria rigidula* rhizome”. The *orange line* represents “*Drynaria sparsisora* rhizome”

While the TPC, FRS, FRP, TFC and BCB assays are measures of primary antioxidant activity, ferrous ion chelating (FIC) activity is a measure of secondary antioxidant activity—the ability to prevent the formation of radicals, such as those generated by the Fenton reaction in the presence of free ferrous ions [31]. Interestingly, the fertile fronds and rhizomes of all three species showed a low ferrous ion chelating activity, with the fertile fronds of *D. rigidula* showing the highest chelating activity at approximately 45 % at 7 mg/mL (Fig. 2). This appears to be a common phenomena amongst ferns, as even those with exceptionally high TPC values exceeding 2500 mg GAE/100 g, such as *Cyathea latebrosa*, *Cibotium barometz* and *Dicranopteris linearis*, have been reported to exhibit poor chelating activity [27]. The phenolic compounds present in the Drynaria samples are likely weak iron chelators, and thus, weak secondary antioxidants.

As shown in Table 2, *D. quercifolia* fronds showed a high tyrosinase inhibition activity (exceeding 50 %) [32], and may therefore have possible commercial applications as a natural tyrosinase inhibitor, such as in the cosmetics industry (skin whitening) and food industry (antibrowning preservation) [5]. Interestingly however, most of the extracts exhibited tyrosinase enhancement activity, effectively acting as tanning agents. While reports on tyrosinase inhibition are more common, a number of extracts are known to increase melanogenesis, including kava rhizomes, lotus flowers and mangosteen leaves [33]. Although phenolic compounds are often tyrosinase inhibitors, several are capable of acting as tyrosinase activity enhancers, such as naringenin [5] and 4′-O-β-d-glucopyranosyl-(1→2)-β-d-glucopyranosyl-quercetin-3-O-β-d-glucopyranosyl-(1→4)-β-d-glucopyranoside [34]. Macrocycles such as cryptand and crown ethers are also capable of enhancing tyrosinase activity by acting as carriers for water molecules, which in turn increases the enzyme’s activity [35]. Tyrosinase enhancement can see application in skin care products designed to reduce UV damage for skin cancer prevention [34], and as a treatment for hypopigmentation [33]. It can also prove useful in the cosmetics industry, for self-tanning [33]. The exact causal agent for this tyrosinase enhancement would require further investigation, particularly in the fronds of *D. sparsisora*, which showed remarkable tyrosinase enhancement activity.

**Table 2:** Tyrosinase inhibition (%) of Drynaria sp fertile fronds and rhizomes (0.5 mg/mL)

Species	Part	Tyrosinase inhibition (%)
*D. quercifolia*	Frond	56.6 ± 5.0
	Rhizome	−27.9 ± 11.1
*D. rigidula*	Frond	24.9 ± 5.4
	Rhizome	−65.0 ± 6.9
*D. sparsisora*	Frond	−153.7 ± 10.1
	Rhizome	−33.7 ± 2.4

Data in mean ± SD (n = 3). Negative values imply tyrosinase enhancement activity

## Conclusion

Of the three fern species screened, *D. quercifolia* fertile fronds exhibited the highest TPC, FRS, FRP and LPI. The fertile fronds of *D. quercifolia* and *D. rigidula* showed significantly higher TPC and FRP when compared to their rhizomes, with *D. quercifolia* fronds also exhibiting significantly higher FRS. *D. sparsisora* rhizomes on the other hand showed similar antioxidant activity with the fronds, with the exception of the rhizome’s FRS being significantly higher than that of the fronds. Interestingly, regardless of the differences in other antioxidant properties, the LPI between the fronds and rhizomes of all three species were comparable at the highest concentration studied. The fronds of *D. quercifolia* showed good tyrosinase inhibition, while the fronds of *D. sparsisora* showed remarkable tyrosinase enhancement. These findings may hopefully provide further impetus into looking at the fronds of other ferns which are commonly used mostly for their rhizomes.
